# Dialogism in publicity discourses of Anglo-American and Chinese universities: A comparative analysis based on Engagement System

**DOI:** 10.1371/journal.pone.0340273

**Published:** 2026-01-02

**Authors:** Lu Zheng

**Affiliations:** School of Humanities, Jilin University, Changchun, China; Dong-A University College of Business Administration, KOREA, REPUBLIC OF

## Abstract

Dialogism serves as a critical characteristic of university publicity discourse, encapsulating the origins of ideas and the communicative intentions inherent in such discourse. While both Chinese and Anglo-American university publicity discourses fall under the same category, they exhibit distinct dialogic approaches. To investigate the similarities and differences in dialogism, this study conducts a comparative analysis of dialogic contraction and dialogic expansion within English and Chinese publicity discourses, utilizing the Engagement System developed by functional linguist Martin J.R. The findings indicate that English discourse predominantly articulates its viewpoints through negative constructions to refute alternative perspectives, whereas Chinese discourse asserts its positions firmly with affirmative statements to limit space for opposing views. In terms of dialogic expansion, English discourse allows room for alternative viewpoints when presenting its own stance, while Chinese discourse directly cites other perspectives to bolster its arguments. This study has theoretical and practical significance. Theoretically, it advances Martin’s Engagement System and offers a replicable protocol for cross-cultural discourse studies. Practically, it guides university communications and provides a template for analysts integrating qualitative and quantitative methods.

## 1. Introduction

With the advancement of higher education in China, the construction of the “Double First-Class” (world-class universities and disciplines) initiative is progressing vigorously. To achieve this goal, universities are continuously committed to updating educational philosophies, recruiting distinguished faculty members, enhancing collaboration with other institutions, and promoting scientific and technological research outcomes. These achievements can be effectively publicized both domestically and internationally through publicity discourses. Publicity discourse is vital to study because it is an important means of external publicity and a window for external communication [1]. Publicity discourse has various types which are used in corresponding domains, such as: society, urban development, economy, culture and education, etc. [2]. Publicity discourse has a wide range of applications, involving many fields, and the scholars have different understandings of publicity discourse, so that the research on publicity discourse is relatively scattered [3–6]. However, a common feature of all forms of publicity discourse is external communication, which underscores the critical role of dialogism within such discourse.

The form of dialogue has been a philosophical method of thinking since Plato’s dialogues and is also evident in Kant’s antinomies. Later, Hegel expanded this approach through dialectics, while Vygotsky further developed the concept of dialogism. However, it was Bakhtin who first formalized dialogue as a method within the philosophy of language. Bakhtin [7] posited that dialogic philosophy falls under the broader category of translinguistics, addressing aspects such as eventality, subjectivity, communication, value evaluation, and dialogism inherent in verbal exchanges. Bakhtin [8] argued that the distinction between monologue and dialogue is not absolute; every dialogue contains elements of monologue, and every monologue can be seen as part of a larger dialogue. Building on this, he introduced the concepts of “monogloss” and “heterogloss”, which eventually led to the formation of his dialogue theory. Bakhtin’s dialogue theory has since become a foundational framework for subsequent scholarly investigations into dialogism [9–17].

Bakhtin’s translinguistic philosophy and dialogism [18] have profoundly influenced Martin, a systemic-functional linguist. Building on Bakhtin’s dialogic thought, Martin conceptualizes “voice” in discourse as a form of discourse intervention and an integral part of the “Appraisal System” [19]. He continued to employ Bakhtin’s concepts of “monogloss” and “heterogloss”, further developing the latter with a heightened focus on dialogic complexity. Martin [20] argues that within the dialogic framework of discourse, there is not only the expansion and exchange of dialogue but also constraints on dialogue and the assertion of independent viewpoints. Consequently, he refined “heterogloss” into two dimensions: “dialogic expansion” and “dialogic contraction”. This refinement led to the formation of the “Engagement System” as a subsystem of his “Appraisal System”. Martin’s “Engagement System” has significantly impacted the publicity discourse analysis, particularly in the study of dialogism in the discourse. In recent years, research on dialogism has been advancing vigorously. To clarify the research framework of this study and highlight its necessity, a review of the major research findings is presented as follows.

## 2. Literature review

### 2.1 Concept of dialogism and related studies

“The notion of dialogism can be defined as the orientation of discourse towards other instances of discourse.” [21]. The exploration of dialogism is mainly embodied in the academic thoughts of Vygotsky, L.S. and Bakhtin, M.M. Both are representative thinkers who accomplished achievements in the discussion of dialogism. The initial purpose of the discussion is to define the distinctive traits of humanness, namely, what is it that distinguishes human from non-human.

The common conclusion is that human have consciousness and dialogue. The two thinkers differ from and complement with each other. According to Eun [22], they arrive at a common conclusion that dialogism is the crucial yardstick distinguishing human from other living beings. Vygotsky [23] and Bakhtin [7] both agree that the multiple voices and utterances exist in human communication and should be studied in social context.

However, the divergence arises when the two thinkers interpret the nature of dialogism. Vygotsky [24] approached the nature of dialogism from the dialectical stance while Bakhtin from a polyphonic view. The aim of Vygotsky’s dialectics is to negotiate the disagreements of two views via a rational dialogue. Unification, systematization and synthesis of multiple voices and consciousness are approaches sought by Vygotsky [25]. Just as White [26] points out, general knowledge and universal truth are attainable. Whereas, Bakhtin resists any types of dialectics and holds that it is impossible for multiple voices and consciousness to merge.

Bakhtin [8] proposes his conception of polyphonic dialogism based on the polyphonic novel written by Dostoevsky. He holds that the discourses of the author do not control those of the characters in the polyphonic novel while the authorial discourses dominate the voices and views of the characters in monologic novels. Based on the study, Bakhtin proposes the terms “heterogloss” and “monogloss” and finally forms his Dialogue Theory. Bakhtin’s Dialogue Theory has emerged as a central framework in the study of discourse and dialogue.

In recent years, numerous studies have employed Bakhtin’s theory to investigate the dialogism of diverse discourses, including publicity discourse, educational discourse, polemic discourse, literary discourse, and political discourse. Regarding publicity discourse, Mende et al. [13] explored print and digital media advertisements through the lens of Bakhtinian polyphonic theory. Their findings indicate that dialogism significantly influences the development of responsive-active readers by fostering an understanding of Alterity. Additionally, Sousa [17] examined posts and comments on Facebook within the framework of dialogism, convergence, participatory culture, and social networks. The study revealed that profiles on social networks often reproduce statements originally elaborated elsewhere. In the context of educational discourse, Kim [11] analyzed classroom dialogues across various subjects. The research concluded that Bakhtin’s concepts of “internally persuasive discourse” and “polyphony” can facilitate the realization of intercultural principles. Moreover, Santana & Marques [15] investigated the contributions of dialogism in reading texts, highlighting its significant impact on cultivating responsive-active readers through Alterity. Concerning polemic discourse, Santulli [16] examined the dialogicity and argumentation in environmental dialogues, concluding that both elements are pervasive in discourse. Based on this, he defined the concept of interdiscourse using Bakhtin’s Dialogism. As a representative study on literary discourse, Busatto & Pereira [9] focused on intertextual and hypertextual strategies in digital narratives, demonstrating that the digital genre enables innovative exploration of readings and cultures and identifying that utterances are unique and unrepeatable, and therefore heterogeneous. Regarding political discourse, Qulebsir-Oukil [14] analyzed linguistic markers in political remarks, identifying their crucial influence on shaping public opinion.

The aforementioned research primarily focuses on Bakhtin’s dialogue theory through the lens of polyphony or heteroglossia, examining intertextuality, heterogeneity, dialogism, and extralinguistic phenomena in discourse. This exploration reflects Bakhtin’s semiotic linguistic ontology and his translinguistic philosophy from various perspectives. However, the studies above only focus on the expansion of dialogue in multiple voices and ignores the contraction of dialogue which is also considered as part of dialogism. In addition, “dialogue” functions as a product of cultural symbols within a specific network system [22,27] and cultural context deeply influenced the dialogic patterns. The integration of “dialogue” and “cultural context” in these studies is inadequate.

### 2.2 Engagement system and dialogism

Functional linguist Martin & White [19] considers “dialogue” as a form of discourse intervention, positioning it as a conjunctive subsystem within the appraisal system network. Thereafter, they introduce Bakhtin’s Dialogue Theory and develops the Engagement System of Appraisal Theory. In Martin’s theory, “heterogoss” and “monogloss” are applied to explore the sources of various voices and dialogism embodied in discourse.

**Fig 1:**
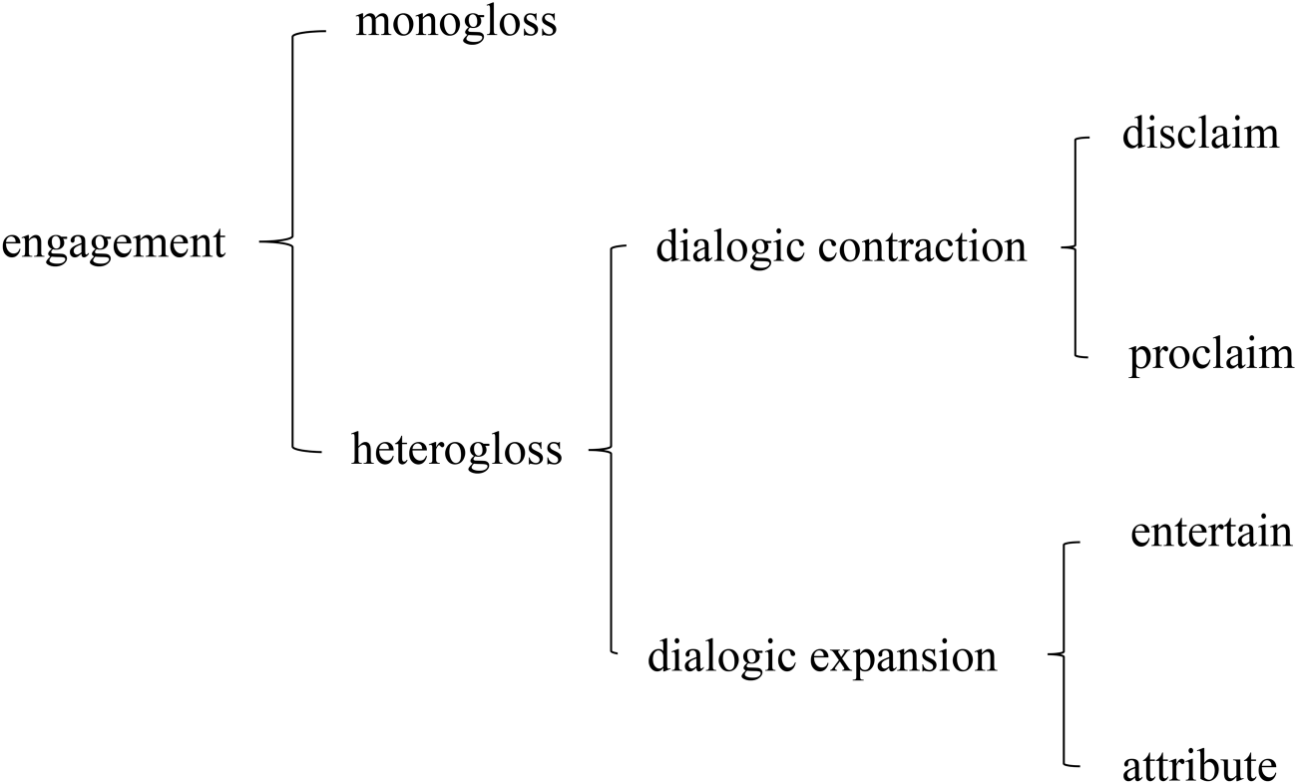
Engagement system.

Engagement refers to the source of appraisal [27]. As one of the three systems of Appraisal Theory developed by Martin, the Engagement System primarily investigates the attitudes, positions, and sources of diverse viewpoints embodied in a discourse, thereby revealing its dialogism. According to Martin & White [19], the objective of the Engagement System is for discourse creators to express their values to readers and establish an alliance with them. Alignment goes beyond mere agreement and it also encompasses tolerance and acceptance of different perspectives [20]. Martin’s Engagement System draws inspiration from Bakhtin’s theory of dialogue. In accordance with Bakhtin [7], speakers employ various linguistic resources to construct a multi-vocal background that conveys propositional views and facilitates communication with others. Speakers introduce other voices through different language resources in varying degrees and manners during dialogues. The schematic representation of Martin’s Engagement System is illustrated in Fig 1.

According to the degree of dialogism, engagement encompasses two aspects: “monogloss”and “heterogloss”. Monogloss represents a direct assertion that implies the correctness and reasonableness of a certain viewpoint without requiring verification or argumentation. It may disregard other differing views as insignificant [19]. This approach excludes dialogue with readers and does not rely on evidence or support from other voices. Consequently, it is highly subjective, placing significant responsibility on the discourse for its held viewpoint [28]. On the other hand, heterogloss tolerates and acknowledges the existence of diverse perspectives by incorporating quotations from others to bolster their own opinions [19,28]. This inclusive stance allows for objectivity in discourse while reducing individual responsibility [19]. The key characteristic of heterogloss lies in its dialogic nature.

The concept of heterogloss can be classified into two categories: “dialogic contraction” and “dialogic expansion” [27]. Dialogic contraction refers to the narrowing of the dialogic space and the limitation of alternative positions and viewpoints [19]. It can further be divided into two subcategories: “disclaim” and “proclaim”. Disclaim is expressed through negation or counter-expectation (concession), while proclaim involves strongly agreeing with a viewpoint, considering it reliable and reasonable, in order to resist other perspectives. On the other hand, dialogic expansion entails expanding the dialogic space within discourse by tolerating diverse opinions and voices to a significant extent [19,29]. This category can also be subdivided into “entertain” and “attribute”. Entertain is used to indicate that a viewpoint may be correct or that the author’s perspective is one among several valid viewpoints. Attribute refers to directly quoting someone else’s words or ideas (including direct quotes and formulaic references) in order to incorporate their viewpoints into the dialogic space. Dialogic contraction and dialogic expansion of heterogloss are two main ways to measure dialogism in publicity discourse [29]. Notably, the degree of dialogism exhibited by dialogic expansion surpasses that demonstrated by dialogic contraction.

However, a key limitation of Martin & White’s [19] original framework is its relative inattention to how genre conventions (institutional discourse templates) shape engagement choices—a gap that this study fills to avoid conflating “cultural differences” with “genre-driven differences”. As Bhatia [30] defines it, “genre conventions” refer to the shared, context-specific patterns of language use that emerge within a particular discourse community (e.g., university publicity) to achieve communicative goals (e.g., promoting the institution, attracting students). In university publicity discourse, these conventions include standardized structures (e.g., “institutional achievements → faculty strengths → student outcomes”) and formulaic expressions (e.g., citing rankings, accreditation bodies) that transcend cultural boundaries.

This study argues that engagement choices in Chinese and Anglo-American university publicity discourse are shaped by two intertwined factors: cultural values and genre conventions—and the Engagement System alone cannot distinguish between the two. For example: A preference for “attribute” (expansion) in Chinese publicity (e.g., “As the State Council emphasizes, our university leads in innovation”) may reflect both “high-context cultural norms” [31]—relying on authoritative voices to build trust—and “genre conventions” (global university publicity often cites government or accreditation bodies). A higher frequency of “proclaim” (contraction) in Anglo-American publicity (e.g., “We offer personalized student support”) may stem from both “low-context cultural norms” (direct expression of institutional strengths) and “genre conventions” (a focus on student-centric value propositions common in Western higher education marketing).

To disentangle these influences, this study integrates the “genre conventions” perspective into its analytical framework: we isolate “culture-specific variations”: Engagement choices that diverge from these universal conventions (e.g., Chinese publicity’s higher reliance on government authorities, Anglo-American publicity’s higher reliance on student testimonials) are attributed to cultural factors, rather than genre. This integration ensures that our comparative analysis of Chinese and Anglo-American discourse does not misattribute genre-driven choices to cultural differences—a critical correction to Martin & White’s [19] framework, which prioritizes interpersonal dialogism over institutional context.

The choice of dialogism as the focus in the current study is due to its inherent connection with publicity discourse, including the publicity discourse of university. Dialogism occurring in publicity language in one social-culture context may manifest itself differently in that of another [32]. This emphasizes the necessity of juxtaposing publicity discourse practices in linguistically distant cultures, such as English and Chinese. As far as the publicity discourse of university is concerned, there has been few attempts made to explore the source of voices in English and Chinese publicity language practice used for publicizing the situation of universities. Therefore, there remains more to be learnt about the similarities and differences in English and Chinese publicity discourses. Based on this, the current study attempts to conduct a comparative study on the dialogism of Anglo-American and Chinese university publicity discourses from the perspective of Engagement System and exert a linguistic-cultural interpretation to provide some alternative insights. More specifically, we mainly aim to answer the following question: How is the dialogism of publicity discourse of university in China different from that in Britain and the United States?

## 3. Methodology

### 3.1 Corpus

University profile is typical publicity discourse of university, which embodies the publicity function of a certain university. For the current study, the profiles of 40 Chinese “double top” universities (First-class universities and disciplines of the world) and those of 40 British and American universities from the top 500 universities in the world QS World University Rankings (2022 edition) are selected from the official website as the Chinese and English corpus for comparative analysis. These chosen institutions are all renowned high-level universities, encompassing various fields such as liberal arts, science, engineering, etc., ensuring their representativeness among similar establishments. The Chinese corpus consists of 2,976 sentences comprising 84,832 characters while the English corpus comprises 1,232 sentences totaling to 18,828 words. To ensure comparability in the comparative analysis, it is crucial to examine the register theory-based comparability between the collected English and Chinese corpora.

### 3.2 Comparability

According to Halliday’s “register theory” [33], language exhibits various forms, known as functional varieties of language, which arise due to changes in situational context. Register encompasses three dimensions: field, tenor, and mode. Field pertains to the communicative theme of discourse. Tenor refers to the social relations and communicative purposes between interacting parties. Mode denotes the channels or media employed in language communication, such as written language or colloquial style.

In terms of field, both English and Chinese discourses serve as university profiles, providing an overview of the institution and facilitating external communication. Concerning tenor, they both aim to establish effective communication between university officials and readers in order to achieve their communicative goals. From the mode perspective, both employ written forms with dialogic implications. They share the same genre, subject matter, communicative intention, and corresponding linguistic structure. Consequently, the selected English and Chinese discourses are comparable.

Furthermore, in terms of content structure, English and Chinese discourses comprise topics such as university property, development history, faculty, facilities, scientific research achievements, awards, and more. Henceforth, the content structure exhibits similarities. Regarding quantity, Chinese discourses tend to be extensive and detailed while English discourses adopt a concise approach with a more general language style. However, to mitigate the issue of length disparity between English and Chinese discourses during data analysis, ANOVA and frequency analysis techniques are employed, consequently ensuring comparability of the selected corpus from a quantitative perspective. Building upon this foundation, further processing of the data is conducted in the subsequent section.

### 3.3 Data analysis

Firstly, this study established a standardized annotation framework for engagement resources to ensure the objectivity and reliability of data extraction. Specifically, this study synthesizes and extracts the conceptions and samples of engagement resources from the representative literature [19,21,25,27–29,34–36] (see Table 1). Based on the conceptions and samples, the engagement resources appearing in the corpus are identified and calculated (To ensure the accurate translation and interpretation of key concepts, a bilingual comparison of core terms in the Engagement System is provided in Appendix 1). Two trained annotators (both with a master’s degree in applied linguistics and prior experience in discourse analysis using the Engagement System) independently participated in the corpus annotation. The annotation process was divided into two phases:

**Table 1:** Conceptions and samples of engagement resources.

Engagement resources	Conceptions	Samples
**Dialogic contraction**	It refers to the contraction of the dialogic space and the restriction of other positions and viewpoints. Disclaim means expressing one’s own viewpoint by negation or counter-expecting a certain opinion; proclaim refers to having a high degree of agreement with a viewpoint.	disclaim: not, do not, rather than, although, 不(not), 不是(do not), 禁止(prohibit), 然而(however), 虽然……但是……(although) proclaim: naturally, of course, obviously, admittedly, I contend, the truth of the matter is, there can be no doubt that, X has demonstrated that, As X has shown, 自然地(naturally), 当然(of course),很明显(obviously), 不得不承认(admittedly), 我赞同(I agree), 事情的真相是……(the truth is……), 毫无疑问 (beyond doubt), etc.
Dialogic expansion	It refers to opening up a dialogic space that is more inclusive of other viewpoints and voices, including entertain and attribute. Entertain is used to express that a viewpoint may be correct, or the author’s viewpoint is one of the correct points. Attribute means quoting some other’s words or ideas to incorporate their viewpoints into dialogic space, including direct quotes (verbatim citation of specific sources) and formulaic references (indirect citation of general/authoritative views).	entertain: it seems, the evidence suggests, apparently, I hear, perhaps, probably, it’s possible, in my view, I suspect that, I believe that, it’s almost certain that, maybe, may, will, must, 好像是(as if), 可能(maybe), 也许(perhaps), 某种迹象表明(the evidence suggests), 从表面上看(on the face of), 据...所知(according to...), 我觉得(in my opinion), 很可能是(probably), etc. attribute: a. direct quotes: X said, X believes, 某人说过(X said); b. formulaic references: in X’s view, according to X, it is said that, it is rumored that, X claims that, 据说(it is said that), 用某人的话来说 (in X’s words), 传言道(it is rumored that), 常言道(as the saying goes),etc.

Pilot Annotation: A subset of the corpus (10% of the total, i.e., 4 Chinese university profiles and 4 Anglo-American university profiles) was selected for pilot annotation. After annotation, the two annotators compared results, discussed discrepancies (e.g., whether “it is likely that” in English should be categorized as “entertain” or “proclaim”), and revised the annotation manual to resolve ambiguous criteria—ensuring consistent understanding of core concepts.

Formal Annotation: The remaining 90% of the corpus was annotated independently by the two annotators. After completing formal annotation, inter-annotator agreement was calculated using Krippendorff’s alpha (a widely used indicator for multi-annotator reliability), yielding a coefficient of 0.87. This value exceeds the generally accepted threshold of 0.80 in linguistic research, confirming that the annotation results were sufficiently reliable and minimizing subjective bias in identifying dialogic features.

Subsequently, the data was analyzed using ANOVA in Excel version 2019 to investigate the extent of dialogism disparity between English and Chinese publicity discourses. Employing ANOVA in Excel enables a scientific assessment of the significance of differences between the two sets of data, with P-value serving as an indicator for determining both the presence and magnitude of such differences [37–38]. Previous studies have utilized ANOVA to examine significant disparities within diverse research domains [37,39]. Our focus lies on various variables’ F value, F critical (crit) value, P-value and η² value within English and Chinese discourses. The F value represents the ratio between mean square (MS) among groups and mean square within groups – a higher ratio indicating greater dissimilarity among relative groups. The F crit value denotes the critical threshold at a given significance level while P-value signifies the probability associated with a specific F value. η² is the effect size, referring to partial eta squared and η² = SSₙₑₜ (sum of squares between groups)/ SSₜₒₜₐₗ (total sum of squares). Regarding difference analysis significance, we concentrate on values presented in Table 3, Table 4, Table 6, Table 7, Table 9, and Table 10 columns. The difference significance is measured as follows:

**Table 2:** Quantity and frequency of heterogloss resources.

Discourse types	Heterogloss	Dialogic contraction	Dialogic expansion
**English discourse**	188	76(40.4%)	112(59.6%)
**Chinese discourse**	240	88(36.7%)	152(63.3%)

**Table 3:** Differential significance of dialogic contraction.

Difference source	SS	df	MS	F	P-value	F crit	η²
**Row**	47.8	39	1.225641	0.627297	0.925122	1.704465	0.009
**Column**	1.8	1	1.8	0.92126	0.343058	4.091279	0.001
**Deviation**	76.2	39	1.953846				
**Amount**	125.8	79					

**Table 4:** Differential significance of dialogic expansion.

Difference source	SS	df	MS	F	P-value	F crit	η²
**Row**	58.8	39	1.507692	0.525	0.97628	1.704465	0.011
**Column**	20	1	20	6.964286	0.011889	4.091279	0.051
**Deviation**	112	39	2.871795				
**Amount**	190.8	79					

If F > F crit, P < 0.01, and η² ≥ 0.06: Extremely significant difference with medium to large effect size.

If F > F crit, P < 0.05, and η² ≥ 0.01: Statistically significant difference with measurable effect size.

If F < F crit, P > 0.05, and η² < 0.01: No significant difference with negligible effect.

Besides, in ANOVA tables (Table 3, Table 4, Table 6, Table 7, Table 9, Table 10), the grouping logic of “Row” and “Column” is explicitly defined to avoid ambiguity:

Row: Represents the “inter-group factor” (40 Chinese universities + 40 Anglo-American universities = 80 samples in total, with 39 degrees of freedom (df) for inter-group variation, calculated as df = number of samples – 1).

Column: Represents the “language type factor” (two groups: Chinese discourse vs. Anglo-American discourse, with 1 degree of freedom (df), calculated as df = number of groups – 1).

Deviation: Represents “within-group variation” (random error, with df = (number of samples – number of groups)), reflecting the dispersion of data within each language group.

Finally, when there is a significant or extremely significant disparity in the utilization of specific linguistic resources between English and Chinese discourses, we conduct further analysis on the frequency of their usage to determine which discourse employs these resources more frequently. Based on the outcomes of the data analysis, we elucidate and discuss the similarities and disparities observed in English and Chinese discourses. In conclusion, this study employs both quantitative and qualitative research methods to undertake a comparative analysis on dialogism. This study did not involve human subjects, animal experiments, or the use of sensitive data requiring ethical approval. Therefore, no specific ethical review and approval were required for this work.

## 4. Results

### 4.1 Overall analysis

From the perspective of “dialogic contraction” and “dialogic expansion” categorized in heterogloss, this study examines the differential significance of dialogism in English and Chinese publicity discourses. Furthermore, it analyzes the frequency of usage when a significant or extremely significant difference emerges, aiming to comprehensively grasp their similarities and differences. The results are presented in Tables 2–4.

The results in Table 3 indicate that the difference between English and Chinese discourse in terms of dialogic contraction is statistically insignificant (F value = 0.92126, F crit value = 4.091279, F < F crit, P-value = 0.343058 > .05, η² = 0.001). The results presented in Table 4 demonstrate a significant distinction between English and Chinese discourses regarding dialogic expansion (F value = 6.964286, F crit value = 4.091279, F > F crit, P-value = 0.011889 > .01 and <.05, η² = 0.051). Furthermore, Table 2 reveals that the frequency of dialogic expansion resources in Chinese discourse (63.3%) surpasses that of English discourse (59.6%), suggesting a higher occurrence rate of dialogic expansion resources in Chinese.

Based on the aforementioned data analysis, it can be observed that the utilization of dialogic contraction resources in both English and Chinese discourses is comparable, whereas that of dialogic expansion resources differs significantly. In both languages, there is a higher frequency of dialogic expansion resources than dialogic contraction resources. Consequently, it can be inferred that discourse in both English and Chinese places emphasis on dialogue by welcoming diverse voices and perspectives while aiming to engage readers. The subsequent section will provide an explicit examination from two angles: dialogic contraction and dialogic expansion.

### 4.2 Dialogic contraction

The phenomenon of dialogic contraction encompasses both “disclaim” and “proclaim”. This study examines the differential significance and frequency of dialogic contraction resources in English and Chinese discourses, as presented in Tables 5–7.

**Table 5:** Quantity and frequency of dialogic contraction resources.

Discourse types	Dialogic contraction	Disclaim	Proclaim
**English discourse**	76	60(78.9%)	16(21.1%)
**Chinese discourse**	88	0(0%)	88(100%)

**Table 6:** Differential significance of disclaim.

Differene source	SS	df	MS	F	P-value	F crit	η²
**Row**	22	39	0.56413	1	0.5	1.704465	0.025
**Column**	45	1	45	79.7723	5.67E11	4.091279	0.506
**Deviation**	22	39	0.56413				
**Amount**	89	79					

**Table 7:** Differential significance of proclaim.

Difference source	SS	df	MS	F	P-value	F crit	η²
**Row**	49.8	39	1.276923	1.45614	0.122511	1.704465	0.033
**Column**	64.8	1	64.8	73.89474	1.54E-10	4.091279	0.433
**Deviation**	34.2	39	0.876923				
**Amount**	148.8	79					

The results presented in Table 6 indicate an extremely significant difference between English and Chinese discourses regarding the use of disclaim resources, with an F value of 79.77273 (F crit = 4.091279, F > F crit), a P-value of 5.67E-11 (<.01) and η² = 0.506. As depicted in Table 5, the frequency of disclaim resources in English discourse is notably higher at 78.9%, while it is absent in Chinese discourse (0%). The results presented in Table 7 demonstrate a substantial disparity between English and Chinese discourses concerning the utilization of proclaim resources, with an F value of 73.89474 (F crit = 4.091279, F > F crit), a P-value of 1.54E-10 (<.01) and η² = 0.433. Regarding the frequency distribution shown in Table 5, English discourse exhibits a significantly lower occurrence rate at only 21.l%, whereas proclaim resources are present throughout all instances of Chinese discourse (100%).

The data analysis above indicates an extremely significant disparity in the utilization of disclaim and proclaim resources between English and Chinese discourses. In Chinese discourse, exclusively proclaim resources are employed, with no presence of disclaim resources. Conversely, in English discourse, there is an absolute advantage in the use of disclaim resources while the utilization of proclaim resources is comparatively disadvantaged. Hence, it can be observed that English and Chinese discourses adopt diametrically opposing approaches when it comes to constraining dialogic space. Chinese discourse excels at directly expressing their own viewpoints, whereas English discourse tends to negate alternative perspectives in order to assert their own stance. The cases are as follows:

(1) a. *The XX College financial aid program requires* ***no*** *contribution from XX families with annual incomes below $65,000.* (disclaim)
b. *We are fun and quirky, elite* ***but not*** *elitist, inventive and artistic, obsessed with numbers, and welcoming to talented people* ***regardless of*** *where they come from.* (disclaim)
(2) XX主动服务国家“一带一路”倡议和文化“走出去”重大战略, **率先提出**“多语种+” 卓越国际化人才培养战略, 创新育人模式, 以内涵建设提升办学水平 (XX takes the initiative to serve the national “Belt and Road” Initiative and the major cultural “going out” strategy, **takes the lead in proposing** the “multilingual +” excellent international talent training strategy, innovates the education model, and improves the educational level by connotation construction) . (proclaim)

In Case (1a), the negative term “no” is employed to refute the notion that families with an annual income below $65,000 contribute funds towards the “college aid program”. This approach negates alternative practices or ideas, thereby highlighting the benevolence of the program and emphasizing the resolute stance of its initiator. In Case (1b), the phrase “but not” is utilized in the first half of the sentence to specify the desired talents sought by the university. The subsequent phrase “regardless of” conveys a welcoming attitude towards talents from diverse backgrounds, showcasing a broad-minded perspective and steadfastly resisting opposing voices and opinions to underscore the university’s unwavering standpoint. In Case (2), XX takes the lead in proposing a remarkable international talent training strategy known as “multilingual +”. This confident and contented presentation significantly restricts alternative viewpoints and objections while compressing the room for dialogue.

Based on the data presented in Table 6 and the aforementioned analysis, it is evident that English discourse places emphasis on employing disclaim resources for dialogic contraction, while Chinese discourse predominantly utilizes proclaim resources. Dialogic contraction serves as a valuable intervention enabling more direct and firm expression of discursive attitudes and values, thereby persuading readers to accept the conveyed views. This approach also fosters a sense of alliance with readers. English discourse employs disclaim resources primarily to refute alternative perspectives, thus preparing readers psychologically before expressing its own viewpoints. These disclaim resources are mainly employed in describing university life (such as regulations regarding campus tour fee collection or public fundraising for university construction), rendering the discourse more vivid and humanized. Conversely, Chinese discourse attaches great importance to utilizing proclaim resources directly conveying university values and ideas (such as development concepts or guiding ideologies), resulting in a more formal, powerful, and convincing discourse.

### 4.3 Dialogic expansion

As mentioned above, dialogic expansion is classified into “entertain” and “attribute”. The significance and frequency of use of dialogic expansion resources in English and Chinese discourses are analyzed from these two perspectives, as illustrated in the following Tables 8–10.

**Table 8:** Quantity and frequency of dialogic expansion resources.

Discourse types	Dialogic expansion	Entertain	Attribute
**English discourse**	112	72 (64.3%)	40 (35.7%)
**Chinese discourse**	152	28 (18.4%)	124 (81.6%)

**Table 9:** Differential significance of entertain.

Difference source	SS	df	MS	F	P-value	F crit	η²
**Row**	30	39	0.769231	0.773196	0.787262	1.704465	0.032
**Column**	24.2	1	24.2	24.32474	1.55E-05	4.091279	0.260
**Deviation**	38.8	39	0.994872				
**Amount**	93	79					

**Table 10:** Differential significance of attribute.

Difference source	SS	df	MS	F	P-value	F crit	η²
**Row**	37.8	39	0.969231	0.512195	0.980157	1.704465	0.019
**Column**	88.2	1	88.2	46.60976	3.7E-08	4.091279	0.443
**Deviation**	73.8	39	1.892308				
**Amount**	199.8	79					

According to Table 9, the F value for entertain resources is 24.32474, exceeding the critical F value of 4.091279 (F > F crit). Additionally, the P-value of 1.55E-05 < .01 and η² = 0.260 indicate an extremely significant difference in the utilization of entertain resources. Furthermore, English discourse exhibits a frequency of entertain resources at 64.3%, while Chinese discourse only shows a frequency of 18.4% (refer to Table 8), suggesting that the occurrence rate of entertain resources is significantly higher in English discourse. Similarly, in terms of attribute resources, as presented in Table 10, the F value is 46.60976 and the critical F value (F crit) is 4.091279. Since F > F crit, the P-value (3.7E-08) <.01 and η² = 0.443, it indicates an extremely significant difference between English and Chinese discourses exists. Furthermore, as illustrated in Table 8, the utilization frequency of attribute resources in English and Chinese discourses are reported as 35.7% and 81.6%, respectively. Notably, the employment rate of attribute resources in Chinese discourse surpasses that observed in English discourse.

Based on the data analysis, a significant disparity is observed in the utilization of entertain and attribute resources between English and Chinese publicity discourses. The frequency of entertain resources in English discourse surpasses that in Chinese discourse, while the frequency of attribute resources falls short compared to Chinese discourse. In contrast, within Chinese discourse, the frequency of attribute resources significantly outweighs that of entertain resources. Consequently, it can be inferred that Chinese discourse places greater emphasis on incorporating diverse viewpoints or voices directly into the dialogic space to enhance objectivity and alleviate certain responsibilities associated with the discourse. Conversely, English discourse prioritizes expressing individual opinions while acknowledging their correctness and accepting alternative perspectives and voices to foster a higher degree of dialogue. The cases are as follows:

(3) *Please note that some Colleges charge an entrance fee and access* ***may*** *be limited at certain times of year– especially during the third term of the year (Easter Term) when many students sit exams.* (entertain)
(4) *1998年5月4日, XX大学百年校庆之际, XX在庆祝XX大学建校一百周年大会上发表讲话, 发出了“****为了实现现代化, 我国要有若干所具有世界先进水平的一流大学****”的号召。(On May 4, 1998, on the occasion of the centennial celebration of XX University, XX delivered a speech at the conference to celebrate the centennial of XX University, and issued the call that “****in order to realize modernization, our country should have some first-class universities with world advanced level****”)* (attribute)

In Case (3), the modal verb “may” is employed to convey the university’s practice of restricting access during specific periods, particularly in the third term. The use of “may” not only conveys a sense of possibility and uncertainty but also serves as a euphemistic expression, demonstrating the school’s openness to considering diverse suggestions or opinions. This fosters an expanded dialogue space and aims to establish rapport with readers. Case (4) directly cites XX’s statements, incorporating them into the dialogue space as a guiding principle for formulating university development goals. This approach enhances objectivity and reasonableness within the discourse while diminishing its dialogic responsibility.

The discourses in both English and Chinese place emphasis on dialogic expansion. While expressing their own viewpoints, English discourse acknowledges the existence of other valid opinions and is open to accepting diverse voices in order to establish a connection with readers and enhance the affinity of the discourse. In contrast, Chinese discourse directly cites authority’ perspectives to support its own arguments, thereby enhancing the persuasiveness and objectivity of the discourse.

## 5. Discussion

The findings indicate that both English and Chinese publicity discourses emphasize the utilization of “dialogic expansion” resources, while recognizing the significance of “dialogic contraction” as an essential element for dialogism. In terms of discourse expansion, English discourse highlights the use of “entertain” resources, whereas Chinese discourse emphasizes the employment of “attribute” resources. Regarding “dialogic contraction”, English discourse tends to employ “disclaim” resources, while Chinese discourse solely relies on “proclaim” resources. Both English and Chinese publicity discourses exhibit differences and similarities concerning spatiality within dialogue. Compared to dialogic contraction, dialogic expansion is more prominently emphasized in both English and Chinese discourses. This similarity can be attributed to their linguistic-cultural origins. The Chinese and Anglo-American populations coexist on the same planet and share similar objective realities. They actively engage in interpersonal communication and maintain conversations with the external world [40]. As educational institutions, whether it be Anglo-American universities or Chinese universities, there is a growing need for increased opportunities for external exchanges and mutual learning.

Dialogic expansion entails the amplification of dialogic space, thereby encompassing a broader range of perspectives and fostering more polyphonic discourse than dialogic contraction. Drawing on Bakhtin’s dialogue theory [41], it is posited that dialogue serves as both the means and the ultimate objective. Martin et al. [32] succinctly summarize Bakhtin’s theory by asserting that “the vitality of language lies in discourse, which derives its value from dialogue, an essential element interwoven within culture”. Consequently, the values embedded within a discourse are reflected through dialogue. Building upon this premise, Martin develops an engagement system that integrates dialogism into discourse. Similar to English and Chinese publicity discourses, it becomes imperative to disseminate university values through meaningful dialogue while showcasing institutional strength and cultural essence – a communicative intention inherent in university publicity discourse. The writing style of a discourse is determined by its communicative intention [33]. In order to effectively convey this intention, universities not only express their culture and values but also incorporate ideas and suggestions from various sectors of society. Therefore, expanding the space for dialogue and forming alliances with readers are key features of publicity discourse. Based on this premise, it is reasonable that both English and Chinese publicity discourses emphasize organizing discourse through dialogic expansion. However, English and Chinese discourses exhibit partiality in their use of “dialogic expansion” resources. English discourse emphasizes the use of “entertain” resources by acknowledging alternative viewpoints while expressing its own views, thereby increasing the affinity within the discourse. On the other hand, Chinese discourse relies more on “attribute” resources to directly quote others’ opinions into the text in order to enhance persuasiveness and objectivity.

Although both English and Chinese publicity discourses primarily focus on dialogic expansion, it is worth noting that dialogic contraction also holds a certain frequency of usage. Dialogic contraction serves as a high-value intervention, enabling the expression of discourse attitudes and values in a more direct and firm manner, thereby persuading readers to accept the conveyed ideas [19]. Consequently, it becomes a means to establish an alliance with readers. The distinction lies in the fact that English discourse predominantly employs negative or anticipatory sentences for disclaiming other viewpoints, thus asserting their own perspectives while preparing readers psychologically. Such disclaim resources are frequently employed when introducing university life (e.g., regulations regarding campus tour fees or public fundraising for university construction), allowing for greater portrayal of vitality and humanization. Conversely, Chinese discourse places significant emphasis on proclaim resources to directly express university development philosophy and guiding ideology, lending formality and power to the discourse while enhancing its convincing and persuasive nature.

Why do English and Chinese discourses, both being university publicity discourses, exhibit these disparities? What is the underlying cause for such variations? Language serves as a vessel for culture, influencing the cognitive processes and linguistic expressions [42]. Different countries or regions possess distinct thinking patterns and worldviews, significantly impacting their respective languages. Anglo-American societies prioritize individual values’ manifestation, whereas Chinese societies emphasize collective societal values [43]. According to Humboldt [42], each language encapsulates a unique worldview and represents one’s experiences of the world. Individuals must comprehend and grasp the characteristics of their own language in order to achieve their objectives. Rather than resorting to the oversimplified binary model of “agrarian vs. maritime civilizations” – a framework that risks cultural essentialism by reducing complex cultural systems to single environmental determinants – this study adopts Scollon & Scollon’s “high-context vs. low-context theory” to interpret the observed linguistic differences. This theory posits that communication styles are shaped by the degree to which contextual information (e.g., cultural norms, institutional authority, shared knowledge) is relied upon to convey meaning, avoiding the overgeneralization of the agrarian-maritime dichotomy [44–46].

Anglo-American cultures are typically categorized as “low-context cultures” [31]. In low-context communication, meaning is primarily conveyed through explicit, verbal language rather than implicit contextual cues. This cultural trait aligns with the emphasis on “entertain” and “disclaim” resources in English university publicity discourse. For “entertain” resources (e.g., “... may be limited at certain times of year” [case 3]), the use of tentative language explicitly acknowledges alternative perspectives, reflecting the low-context preference for transparency and negotiability in communication. For “disclaim” resources (e.g., “requires no contribution from...” [case 1a], “elite but not elitist” [case 1b]), negative or contrastive linguistic forms directly narrow the dialogic space while clearly articulating the university’s stance – a practice consistent with low-context cultures’ focus on direct, unambiguous expression. Such linguistic choices also resonate with the “analytical thinking” rooted in Anglo-American cultural contexts: by scrutinizing potential counterarguments (via disclaim) or acknowledging possible limitations (via entertain), the discourse demonstrates a commitment to rational negotiation rather than one-sided assertion.

In contrast, Chinese culture is widely recognized as a “high-context culture” [31], where meaning is heavily dependent on implicit contextual factors such as institutional authority, shared cultural values, and hierarchical relationships. This characteristic directly explains the prominence of “attribute” and “proclaim” resources in Chinese university publicity discourse. For “proclaim” resources (e.g., “率先提出‘多语种 +’卓越国际化人才培养战略” [leading in proposing the strategy of ‘multilingual+’ for cultivating outstanding international talents] [case 2]), direct, unqualified assertions of the university’s achievements or philosophies draw on the high-context expectation that institutional prestige (a key contextual cue) will lend credibility to the claim. More importantly, the frequent use of “attribute” resources in Chinese discourse – such as quoting authoritative voices (e.g., “... 发出了‘为了实现现代化, 我国要有若干所具有世界先进水平的一流大学’的号召” [...raised a call for ‘To realize modernization, our country must have a number of first-class universities with advanced world levels’] [case 4]) – is closely tied to the authoritative demands inherent in Chinese university promotional texts. In high-context Chinese society, invoking external authorities (e.g., national policies, government leaders, or institutional mandates) serves two critical functions: first, it leverages the high-context trust in hierarchical authority to enhance the discourse’s persuasiveness without requiring extensive explicit justification; second, it aligns the university’s image with national or institutional goals, reinforcing the collective values that are central to high-context communication. This reliance on authoritative “attribute” resources also reflects the cultural emphasis on “harmony and hierarchy”: by citing recognized authorities, the discourse avoids confrontational negotiation (common in low-context cultures) and instead positions the university as a responsible participant in the broader institutional and national framework.

Additionally, Hyland’s “disciplinary culture” perspective further supplements this analysis by highlighting the role of institutional norms in shaping linguistic choices [47], beyond general cultural differences. For instance, the global “university publicity genre” universally emphasizes the need to balance institutional pride with audience engagement – a disciplinary norm that explains why both English and Chinese discourses prioritize dialogic expansion. However, the *specific* resources chosen to achieve this balance (“entertain”/“disclaim” vs. “attribute”/“proclaim”) are shaped by the intersection of disciplinary conventions and high/low-context cultural traits. In Chinese contexts, disciplinary expectations for institutional legitimacy intersect with high-context reliance on authority, amplifying the use of “attribute” resources; in Anglo-American contexts, disciplinary values of transparency intersect with low-context directness, favoring “entertain” and “disclaim” resources.

Based on the aforementioned discussion, Table 11 summarizes both research findings and their cultural underpinnings, updated to reflect the high-context/low-context framework.

**Table 11:** The research results and cultural roots.

		English discourse	Chinese discourse
**Similarities**	Results	More emphasis on “dialogic expansion” than “dialogic contraction”	
	Cultural roots	English: Shared objective world; shared communicative intention of university publicity (disciplinary culture); low-context preference for transparent dialogue Chinese: Shared objective world; shared communicative intention of university publicity (disciplinary culture); high-context reliance on contextual credibility	
**Differences**	Results	More emphasis on “entertain” and “disclaim”	More emphasis on “attribute” and “proclaim”
	Cultural roots	Low-context culture (direct, negotiable expression); analytical thinking focused on rational scrutiny	High-context culture (authority-dependent, implicit meaning); emphasis on institutional authority (driving “attribute” resource use)

The aforementioned analysis of the similarities and disparities between English and Chinese publicity discourses reveals that linguistic variations are intricately intertwined with their cultural backgrounds, particularly the high-context/low-context dimensions, as well as disciplinary norms of university publicity. Culture provides the foundational context for language use, while language serves as the carrier of cultural values and communication styles. As Malinowski [48] points out, language is deeply rooted in the culture and customs of an ethnic group and should be studied within broader contextual frameworks. To fully understand the linguistic choices in university publicity discourse, it is essential to examine not only surface-level cultural labels but also the nuanced ways in which cultural contexts (e.g., high/low-context communication) and institutional demands (e.g., authoritative expectations in Chinese universities) shape discourse strategies.

## 6. Conclusion

Dialogism serves as a cornerstone of Anglo-American and Chinese university publicity discourses, functioning to disseminate institutional values, showcase strengths and cultural traits, and foster connections with readers through inclusive dialogue. Guided by Martin’s Engagement System, this study adopts a mixed-methods approach (quantitative + qualitative) to compare Chinese and Anglo-American corpora, yielding core findings that converge on both shared tendencies and distinct cultural-specific patterns: Both discourses prioritize dialogic expansion over dialogic contraction to embrace diverse perspectives, yet they diverge in resource deployment—English discourse leans on “entertain” (tentative language to accommodate alternative views) and “disclaim” (negation to refute opposing stances), while Chinese discourse relies heavily on “attribute”(citing authoritative voices to bolster credibility) and “proclaim” (affirmative assertions to assert positions). Rooted in Scollon & Scollon’s“high-context vs. low-context”theory, these differences are attributed to cultural underpinnings: Anglo-American low-context culture emphasizes transparent, negotiable expression, while Chinese high-context culture values institutional authority and implicit credibility.

This study delivers both theoretical and practical contributions. Theoretically, it advances Martin’s Engagement System by integrating ANOVA with effect size (η²) to quantify the magnitude of dialogic differences, and supplements the framework with Scollon & Scollon’s cultural theory to disentangle cultural and genre-driven variations—addressing the scattered nature of existing publicity discourse research. It also establishes a replicable protocol for cross-cultural dialogic analysis, including explicit ANOVA grouping logic and a bilingual core term glossary. Practically, it offers targeted guidance for university communications: Chinese universities can enhance international audience affinity by balancing “attribute” resources with “entertain” elements, while Anglo-American institutions may strengthen credibility in high-context markets through strategic “attribute” use. Additionally, it aids cross-cultural education practitioners in decoding dialogic cues to reduce misunderstandings, and provides a template for analysts integrating qualitative and quantitative methods.

This research has inherent limitations.

To ensure corpus representativeness and purity, the study focused exclusively on high-level universities (Chinese “Double First-Class” and Anglo-American QS Top 500 institutions), excluding ordinary universities whose publicity discourses may exhibit distinct characteristics. This limits the generalizability of findings. Future research should expand the corpus to include diverse university types and publicity discourse genres, broadening the comparative scope to refine and extend the study’s conclusions .

## Supporting information

S1 File Bilingual comparison of core terms. (DOCX)
