# Supplementary material for: Dialogism in publicity discourses of Anglo-American and Chinese universities: A comparative analysis based on Engagement System
Source: PLoS One. 2026 Jan 2;21(1):e0340273. doi: 10.1371/journal.pone.0340273 (PMC12758714; doi:10.1371/journal.pone.0340273)
Supplement: S1 File — (DOCX) [file pone.0340273.s001.docx]

Table of Engagement Resource Quantities in English Publicity Discourses

| **University** | **Dialogic Contraction** | **Dialogic Expansion** |
| --- | --- | --- |
| 1a | 2 | 3 |
| 2a | 2 | 1 |
| 3a | 4 | 2 |
| 4a | 2 | 3 |
| 5a | 2 | 1 |
| 6a | 2 | 0 |
| 7a | 5 | 5 |
| 8a | 0 | 6 |
| 9a | 0 | 3 |
| 10a | 0 | 4 |
| 11a | 2 | 6 |
| 12a | 1 | 3 |
| 13a | 2 | 4 |
| 14a | 0 | 1 |
| 15a | 0 | 3 |
| 16a | 2 | 2 |
| 17a | 1 | 6 |
| 18a | 5 | 3 |
| 19a | 2 | 4 |
| 20a | 2 | 2 |
| 21a | 2 | 4 |
| 22a | 2 | 2 |
| 23a | 2 | 1 |
| 24a | 2 | 2 |
| 25a | 2 | 5 |
| 26a | 1 | 2 |
| 27a | 3 | 2 |
| 28a | 2 | 5 |
| 29a | 2 | 2 |
| 30a | 2 | 2 |
| 31a | 2 | 4 |
| 32a | 1 | 2 |
| 33a | 3 | 3 |
| 34a | 2 | 0 |
| 35a | 3 | 2 |
| 36a | 3 | 3 |
| 37a | 0 | 3 |
| 38a | 2 | 2 |
| 39a | 2 | 2 |
| 40a | 2 | 2 |

Table of Engagement Resource Quantities in Chinese Publicity Discourses

| **University** | **Dialogic Contraction** | **Dialogic Expansion** |
| --- | --- | --- |
| 1b | 0 | 3 |
| 2b | 1 | 3 |
| 3b | 1 | 3 |
| 4b | 4 | 6 |
| 5b | 3 | 5 |
| 6b | 2 | 5 |
| 7b | 1 | 1 |
| 8b | 3 | 4 |
| 9b | 4 | 3 |
| 10b | 3 | 5 |
| 11b | 1 | 2 |
| 12b | 0 | 4 |
| 13b | 2 | 4 |
| 14b | 3 | 5 |
| 15b | 4 | 4 |
| 16b | 1 | 6 |
| 17b | 2 | 2 |
| 18b | 2 | 3 |
| 19b | 5 | 4 |
| 20b | 2 | 4 |
| 21b | 2 | 3 |
| 22b | 0 | 4 |
| 23b | 0 | 2 |
| 24b | 5 | 4 |
| 25b | 2 | 6 |
| 26b | 1 | 5 |
| 27b | 2 | 2 |
| 28b | 4 | 3 |
| 29b | 3 | 3 |
| 30b | 3 | 5 |
| 31b | 3 | 4 |
| 32b | 1 | 2 |
| 33b | 1 | 2 |
| 34b | 1 | 7 |
| 35b | 2 | 6 |
| 36b | 3 | 4 |
| 37b | 2 | 3 |
| 38b | 2 | 2 |
| 39b | 3 | 3 |
| 40b | 4 | 6 |

Table of Dialogic Contraction Quantities in English Publicity Discourses

| **University** | **Disclaim** | **Proclaim** |
| --- | --- | --- |
| 1a | 2 | 0 |
| 2a | 2 | 0 |
| 3a | 4 | 0 |
| 4a | 1 | 1 |
| 5a | 2 | 0 |
| 6a | 2 | 0 |
| 7a | 3 | 2 |
| 8a | 0 | 0 |
| 9a | 0 | 0 |
| 10a | 0 | 0 |
| 11a | 2 | 0 |
| 12a | 0 | 1 |
| 13a | 1 | 1 |
| 14a | 0 | 0 |
| 15a | 0 | 0 |
| 16a | 2 | 0 |
| 17a | 1 | 0 |
| 18a | 4 | 1 |
| 19a | 1 | 1 |
| 20a | 2 | 0 |
| 21a | 1 | 1 |
| 22a | 2 | 0 |
| 23a | 2 | 0 |
| 24a | 1 | 1 |
| 25a | 2 | 0 |
| 26a | 1 | 0 |
| 27a | 3 | 0 |
| 28a | 0 | 2 |
| 29a | 2 | 0 |
| 30a | 1 | 1 |
| 31a | 1 | 1 |
| 32a | 1 | 0 |
| 33a | 2 | 1 |
| 34a | 2 | 0 |
| 35a | 2 | 1 |
| 36a | 3 | 0 |
| 37a | 0 | 0 |
| 38a | 2 | 0 |
| 39a | 2 | 0 |
| 40a | 1 | 1 |

Table of Dialogic Contraction Quantities in Chinese Publicity Discourses

| **University** | **Disclaim** | **Proclaim** |
| --- | --- | --- |
| 1b | 0 | 0 |
| 2b | 0 | 1 |
| 3b | 0 | 1 |
| 4b | 0 | 4 |
| 5b | 0 | 3 |
| 6b | 0 | 2 |
| 7b | 0 | 1 |
| 8b | 0 | 3 |
| 9b | 0 | 4 |
| 10b | 0 | 3 |
| 11b | 0 | 1 |
| 12b | 0 | 0 |
| 13b | 0 | 2 |
| 14b | 0 | 3 |
| 15b | 0 | 4 |
| 16b | 0 | 1 |
| 17b | 0 | 2 |
| 18b | 0 | 2 |
| 19b | 0 | 5 |
| 20b | 0 | 2 |
| 21b | 0 | 2 |
| 22b | 0 | 0 |
| 23b | 0 | 0 |
| 24b | 0 | 5 |
| 25b | 0 | 2 |
| 26b | 0 | 1 |
| 27b | 0 | 2 |
| 28b | 0 | 4 |
| 29b | 0 | 3 |
| 30b | 0 | 3 |
| 31b | 0 | 3 |
| 32b | 0 | 1 |
| 33b | 0 | 1 |
| 34b | 0 | 1 |
| 35b | 0 | 2 |
| 36b | 0 | 3 |
| 37b | 0 | 2 |
| 38b | 0 | 2 |
| 39b | 0 | 3 |
| 40b | 0 | 4 |

Table of Dialogic Expansion Quantities in English Publicity Discourses

| **University** | **Entertain** | **Attribute** |
| --- | --- | --- |
| 1a | 2 | 1 |
| 2a | 1 | 0 |
| 3a | 2 | 0 |
| 4a | 2 | 1 |
| 5a | 1 | 0 |
| 6a | 0 | 0 |
| 7a | 1 | 4 |
| 8a | 5 | 1 |
| 9a | 2 | 1 |
| 10a | 2 | 2 |
| 11a | 4 | 2 |
| 12a | 2 | 1 |
| 13a | 2 | 2 |
| 14a | 1 | 0 |
| 15a | 2 | 1 |
| 16a | 2 | 0 |
| 17a | 4 | 2 |
| 18a | 2 | 1 |
| 19a | 2 | 2 |
| 20a | 2 | 0 |
| 21a | 2 | 2 |
| 22a | 2 | 0 |
| 23a | 1 | 0 |
| 24a | 2 | 0 |
| 25a | 3 | 2 |
| 26a | 2 | 0 |
| 27a | 2 | 0 |
| 28a | 2 | 3 |
| 29a | 2 | 0 |
| 30a | 1 | 1 |
| 31a | 2 | 2 |
| 32a | 1 | 1 |
| 33a | 0 | 3 |
| 34a | 0 | 0 |
| 35a | 2 | 0 |
| 36a | 2 | 1 |
| 37a | 3 | 0 |
| 38a | 2 | 0 |
| 39a | 0 | 2 |
| 40a | 0 | 2 |

Table of Dialogic Expansion Quantities in Chinese Publicity Discourses

| **University** | **Entertain** | **Attribute** |
| --- | --- | --- |
| 1b | 2 | 1 |
| 2b | 0 | 3 |
| 3b | 1 | 2 |
| 4b | 0 | 6 |
| 5b | 0 | 5 |
| 6b | 1 | 4 |
| 7b | 1 | 0 |
| 8b | 1 | 3 |
| 9b | 0 | 3 |
| 10b | 2 | 3 |
| 11b | 0 | 2 |
| 12b | 2 | 2 |
| 13b | 1 | 3 |
| 14b | 1 | 4 |
| 15b | 0 | 4 |
| 16b | 0 | 6 |
| 17b | 0 | 2 |
| 18b | 1 | 2 |
| 19b | 0 | 4 |
| 20b | 1 | 3 |
| 21b | 0 | 3 |
| 22b | 0 | 4 |
| 23b | 0 | 2 |
| 24b | 0 | 3 |
| 25b | 2 | 4 |
| 26b | 2 | 3 |
| 27b | 0 | 2 |
| 28b | 0 | 3 |
| 29b | 1 | 2 |
| 30b | 2 | 3 |
| 31b | 0 | 4 |
| 32b | 0 | 2 |
| 33b | 0 | 2 |
| 34b | 2 | 5 |
| 35b | 0 | 6 |
| 36b | 1 | 3 |
| 37b | 1 | 3 |
| 38b | 0 | 2 |
| 39b | 1 | 2 |
| 40b | 2 | 4 |

Appendix 1. Bilingual comparison of core terms.

| **English term** | **Chinese translation** |
| --- | --- |
| Engagement System | 介入系统 |
| Dialogic Contraction | 对话收缩 |
| Disclaim | 否认 |
| Proclaim | 宣称 |
| Dialogic Expansion | 对话扩展 |
| Entertain | 引发 |
| Attribute  (including direct quotes and formulaic references) | 摘引  （直接引用与程式化引用均属此类） |
| Monogloss | 单声 |
| Heterogloss | 多声 |
